# Cytomegalovirus drives Vδ2^neg^ γδ T cell inflation in many healthy virus carriers with increasing age

**DOI:** 10.1111/cei.12297

**Published:** 2014-04-24

**Authors:** A Alejenef, A Pachnio, M Halawi, S E Christmas, P A H Moss, N Khan

**Affiliations:** * Department of Clinical Infection, Microbiology and Immunology, Institute of Infection and Global Health, University of LiverpoolLiverpool, UK; † Schools of Cancer Sciences, University of BirminghamBirmingham, UK; ‡ Immunity and Infection, University of BirminghamBirmingham, UK

**Keywords:** γδ T-cells, cytomegalovirus, old age

## Abstract

Cytomegalovirus (CMV) usually causes lifelong asymptomatic infection, but over time can distort immune profiles. Recent reports describe selective expansion of Vδ2^neg^ γδ T cells in healthy and immunocompromised CMV carriers. Having shown previously that virus-specific CD8^+^ and CD4^+^ T cell responses are increased significantly in elderly CMV carriers, probably driven by chronic stimulation, we hypothesized that Vδ2^neg^ γδ T cells may also be expanded with age. Our results show that Vδ2^neg^ γδ T cells are increased significantly in CMV-seropositive healthy individuals compared to CMV-seronegative controls in all age groups. The differences were most significant in older age groups (*P* < 0·0001). Furthermore, while Vδ2^neg^ γδ T- cells comprise both naive and memory cells in CMV-seronegative donors, highly differentiated effector memory cells are the dominant phenotype in CMV carriers, with naive cells reduced significantly in numbers in CMV-seropositive elderly. Although phenotypically resembling conventional CMV-specific T cells, Vδ2^neg^ γδ T cells do not correlate with changes in magnitude of CMV-specific CD4^+^ or CD8^+^ T cell frequencies within those individuals, and do not possess *ex-vivo* immediate effector function as shown by CMV-specific CD4^+^ and CD8^+^ T cells. However, after short-term culture, Vδ2^neg^ γδ T cells demonstrate effector T cell functions, suggesting additional requirements for activation. In summary, Vδ2^neg^ γδ T cells are expanded in many older CMV carriers, demonstrating a further level of lymphocyte subset skewing by CMV in healthy individuals. As others have reported shared reactivity of Vδ2^neg^ γδ T cells towards tumour cells, the composition of γδ T cell subsets may also have implications for risk of developing cancer in elderly people.

## Introduction

Cytomegalovirus (CMV) establishes a lifelong usually asymptomatic infection in immunocompetent individuals [1], which is associated with profound effects on the host immune repertoire [2]. We and others have shown that CMV drives massive oligoclonal expansions of both CD4^+^ and/or CD8^+^ virus-specific memory T cells in healthy carriers which increase with age [3–6], a process termed as memory inflation [4]. These T cell responses are frequently more than 1% of the respective subset in young virus carriers, and often exceed 10% of CD4^+^/CD8^+^ T cells in the elderly [5,6]. CMV-specific T cells are predominantly CD28^low^ effector memory T cells (T_em_) [7], producing high amounts of proinflammatory cytokines such as interferon (IFN)-γ and tumour necrosis factor (TNF)-α. Such high frequencies of T_em_ cells may be useful for limiting viral replication, but could also induce greater inflammation that is damaging at the tissue level [8]. This accumulation of CD28^low^ T_em_ cells is also considered a marker of immunosenescence, the deterioration of immune function with age [9].

While CD4^+^ and CD8^+^ T cell responses have been characterized in great detail [10–13], there is growing evidence that non-conventional T cells expressing gamma-delta (γδ) T receptor cells (TCR) are important in protection against CMV. Unlike αβ (CD4^+^/CD8^+^) T cells, γδ T cells are major histocompatibility complex (MHC)-unrestricted in their antigen recognition and normally constitute a minority of circulating T cells. However, γδ T cells are expanded or activated in infections and in malignancy [14–18], and also differentiate into memory cells much earlier in life than αβ T cells, indicating a major role in responding against pathogenic insult from birth [19]. Dechanet-Merville and colleagues have shown that γδ T cells are considerably expanded (reaching 40% of circulating T cells) following primary CMV infection in CMV-seronegative transplant recipients of kidneys from CMV-seropositive donors [20–22]. Early γδ T cell reconstitution correlated with improved control of CMV replication, and the expansions were composed of either Vδ1^pos^ or Vδ3^pos^ cells, but not Vδ2^pos^ cells [20]. Similar Vδ2^neg^ γδ T cell expansions have also been reported very recently in CMV-infected allogeneic stem cell transplant patients and in CMV-infected fetuses [23,24].

The selective expansion of Vδ2^neg^ γδ T cells (Vδ1^pos^ and Vδ3^pos^) in CMV-infected hosts implies that these γδ T cells are involved in immunity. Expanded Vδ2^neg^ γδ T cell lines specifically secrete cytokines and demonstrate cytotoxicity after incubation with CMV-infected target cells *in vitro*, but not uninfected targets or target cells infected with other herpesviruses [25]. It has been shown recently that Vδ2^neg^ γδ T cells are also expanded in healthy CMV carriers [26] but it is unclear whether, like CMV-specific CD4^+^ and CD8^+^ T cells, Vδ2^neg^ γδ T cells also expand with age. The persistent nature of CMV infection is thought to drive CD4^+^/CD8^+^ T cell memory inflation over time. Thus, we aimed to test our hypothesis that Vδ2^neg^ γδ T cell expansions occur more frequently in CMV carriers of older age.

## Materials and methods

### Study volunteers

A total of 255 healthy adult volunteers, aged 20–85 years, and two non-immunocompromised patients diagnosed with symptomatic primary CMV infection formally consented to donate blood samples for the study. Ethical approval was obtained from local Adult Research Ethics Committees (REC reference 2K/175 and 09/H1005/51). CMV status was determined using plasma samples with a commercial CMV immunoglobulin (Ig)G enzyme-linked immunosorbent assay (ELISA) kit (Biocheck Inc., Foster City, CA, USA). Diamedix herpes simplex virus (HSV) IgG and varicella zoster virus (VZV) IgG kits (Launch Diagnostics, Longfield, UK) was used for HSV and VZV seropositivity and viral capsid antigen (VCA) staining had been performed previously for Epstein–Barr virus (EBV) seropositivity. Absolute lymphocyte counts were determined by the Blood Sciences Department at the Royal Liverpool University Hospital NHS Trust.

### Antibodies and flow cytometry

Peripheral blood mononuclear cells (PBMC) were isolated from donor blood and resuspended in fluorescence activated cell sorter (FACS) buffer [phosphate-buffered saline (PBS) containing 0·1% bovine serum albumin (BSA) and 2 mM ethylenediamine tetraacetic acid (EDTA)], and then incubated with different combinations of monoclonal antibodies (mAb) for 20 min at 4°C at the manufacturer's recommended concentrations. The following mAb were used: TCR-γδ-allophycocyanin (APC), TCR-αβ-phycoerythrin (PE), Vδ2-PE, lymphocyte function-associated antigen 1 (LFA-1)-fluorescein isothiocyanate (FITC), CD4-APC (all purchased from BD Biosciences, San Jose, CA, USA), CD8-PE (Dako, Glostrup, Denmark), Vδ1-FITC (Thermo Fisher Scientific, Loughborough, UK), Vδ2-peridinin chlorophyll (PerCP), CD45RA-Alexa-Fluor 700, CD45RA-PerCP-cyanin (Cy)5.5 and CD28-PerCP-Cy5.5 (all from Cambridge Bioscience, Cambridge, UK), CD27-APC eFluor780 (eBioscience, San Diego, CA, USA), CD57-FITC (AbD Serotec, Kidlington, UK) and CCR7-FITC (R&D Systems, Minneapolis, MN, USA). Intracellular staining was performed with perforin-FITC, granzyme B-PE, IFN-γ-PerCP-Cy5.5 and TNF-α-FITC (all from BD Biosciences). Analysis was performed using a Becton Dickinson FACSCalibur or LSR II flow cytometer. Data were analysed later using Win MDI 2.8 software (The Scripps Institute: http://facs.scripps.edu/software.html) and/or diva software (BD Biosciences).

### Assays of T cell function

CMV-specific CD4^+^ and CD8^+^ T cells were detected as described elsewhere [5,27]. Briefly, PBMC were stimulated for 6 h at 37°C (5% CO_2_) with CMV or mock lysates, or with a cocktail of synthetic peptides (purchased from Invitrogen, Carlsbad, CA, USA) representing published immunodominant human leucocyte antigen (HLA) class I-restricted CMV epitopes from six viral antigens (see [28]). Brefeldin A was added (10 μg/ml final concentration) after 1 h of incubation. Cells were then stained for surface markers and afterwards for intracellular cytokines. Appropriate isotype controls were used for each test.

γδ T cell functional assays involved enrichment of PBMC for γδ T cells using a TCR-γδ T cell isolation kit (Miltenyi Biotec, Bergisch Gladbach, Germany), as per the manufacturer's instructions. In some cases Vδ2 cell depletion was also carried out. γδ T cell lines were generated by co-culture of enriched γδ T cells with irradiated phycohaemagglutinin (PHA)-activated allogeneic PBMC and stimulation with 30 ng/ml anti-CD3 (OKT3; Cambridge Bioscience Ltd) and 100 U/ml of recombinant interleukin 2 (rIL-2) (Peprotech, London, UK). T cell lines were maintained in medium supplemented with 100 U/ml of rIL-2 for up to 4 weeks. Unmanipulated or cultured cells were co-incubated with uninfected human fetal foreskin fibroblasts (HFFF) or HFFF infected for 3–4 days with the AD169 strain of CMV (at multiplicity of infection 1:1). After 6 h at 37°C (with brefeldin A added at 1 h), PBMC were removed and washed before staining for surface markers and cytoplasmic IFN-γ and TNF-α. In parallel experiments, tubes were incubated with FITC-conjugated anti-CD107a (BD Biosciences) at the beginning of the incubation, to determine degranulation as a consequence of stimulation.

T cell lines were also tested for IFN-γ secretion using supernatants taken from overnight-stimulated (with CMV-infected or non-infected fibroblasts) cultures by ELISA (eBioscience) in accordance with the manufacturer's recommended protocol. Blocking assays were performed by preincubating effector cells with anti-TCR-Vδ1, anti-TCR-Vδ2 or mouse isotype control mAb. For positive controls, cells were stimulated with 20 ng/ml PMA and 1 μg/ml ionomycin (both from Sigma, Poole, UK).

### Statistical analyses

These were performed with Graphpad Prism software (GraphPad Software Inc., La Jolla, CA, USA). The Mann–Whitney *U*-test was applied with 95% confidence intervals to test differences in γδ T cell frequencies between different donor groups. The non-parametric Spearman's rank correlation coefficient was used to assess correlations between different T cell subset frequencies. All *P*-values were two-tailed, and for multiple comparisons subjected to Holm–Bonferroni correction.

## Results

### γδ T cell subsets are skewed by CMV carriage in older individuals

**Fig. 1:**
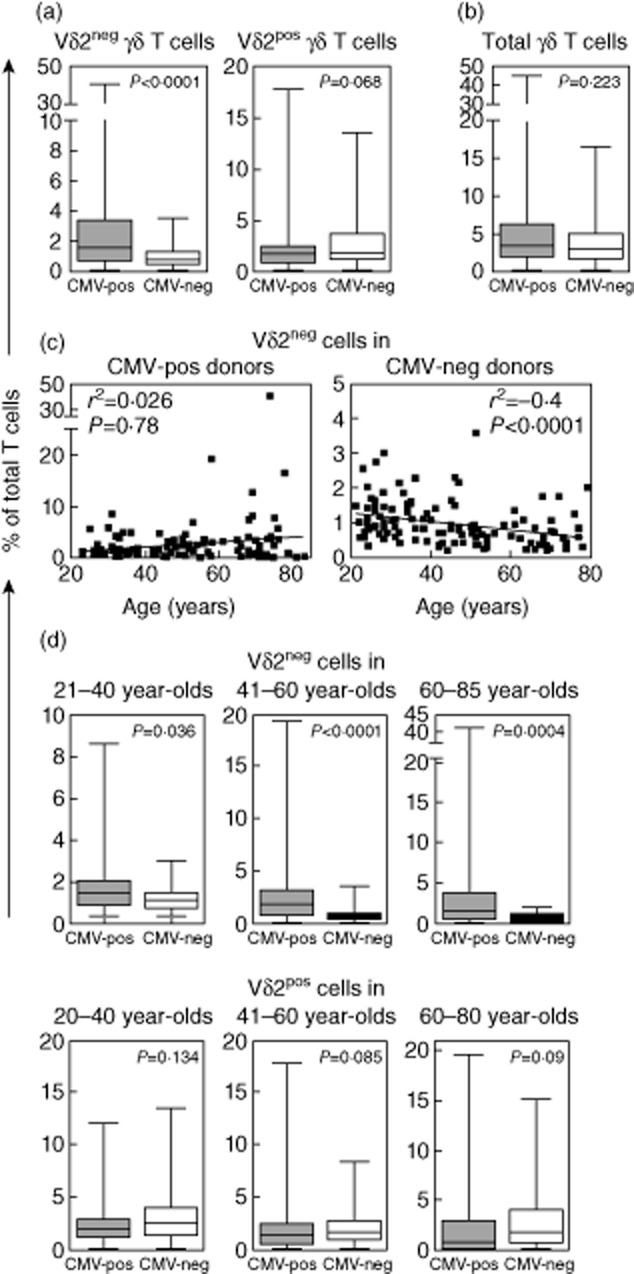
γδ T cell subsets in healthy donors. Charts summarizing the γδ T cell staining results from 255 healthy donors are shown for Vδ2^pos^ and Vδ2^neg^ γδ T cells (a) and total γδ T cells (b). Vδ2^neg^ γδ T cell frequencies with increasing age in cytomegalovirus (CMV)-seropositive and CMV-seronegative donors (c). Comparison of Vδ2^pos^ and Vδ2^neg^ γδ T cells between CMV-seropositive and CMV-seronegative donors in each of the defined age groups (d). Values on the *y*-axis indicate the percentage of total T lymphocytes represented by each subset. *P*-values are shown above each plot with 95% confidence intervals applied.

Our initial investigation of γδ T cells in 255 healthy volunteers (125 CMV-seropositives/130 CMV-seronegatives) aged 21–85 years showed considerable variation in frequency of different γδ T cell subsets in blood. In some individuals Vδ1^pos^ cells were the major γδ type, while in others Vδ2^pos^ cell expansions were observed (see representative examples in Supporting information, Fig. S1). We could not stain directly for Vδ3^pos^ γδ T cells (due to lack of specific mAb), but as they were also expanded in a small number of individuals we measured the total Vδ2^neg^ γδ population to include for Vδ3^pos^ cells. Overall, Vδ2^neg^ γδ T cells were significantly higher (*P* < 0·0001) in CMV-seropositive donors than in CMV-seronegative donors (see Fig. 1a). This coincided with reduced Vδ2^pos^ γδ T cells in CMV carriers, but was not statistically significant (Fig. 1a). However, the total γδ T cell frequency in CMV-seropositive and CMV-seronegative donors was very similar (Fig. 1b). To confirm that this effect was CMV-associated, we tested for other human herpesviruses, HSV-1/2, EBV and VZV. Statistical analysis did not show any significant difference in γδ T cell subsets between seropositive and seronegative donors for these viruses (data not shown), in agreement with work published by others [26].

We then examined if Vδ2^neg^ γδ T cells increased with age (see Fig. 1c). A number of middle- and older-aged donors had Vδ2^neg^ γδ T cell expansions approaching 10% (or more) of all T cells, with the highest observed frequency at 41% of all T cells in one healthy elderly donor; findings that are very similar to that of increased CMV-specific CD4^+^ and CD8^+^ T cells in healthy elderly virus carriers. However, the increase in Vδ2^neg^ cells with age was not statistically significant (*P* = 0·78). Interestingly, there was a significant reduction of Vδ2^neg^ cells in the CMV-seronegative group with age (*P* < 0·0001). Further analysis within separate age groups termed hereafter as young, aged 21–40 years (*n* = 97), middle-aged, aged 41–60 years (*n* = 83) and elderly, aged 61–85 years (*n* = 75), showed that Vδ2^neg^ γδ T cells were significantly higher in CMV carriers of all age groups when compared with age-matched CMV-seronegative donors, both as frequency of total T cells and as the absolute number of cells (see Table 1). In contrast, Vδ2^pos^ γδ T cells were not significantly different between CMV-seropositive and CMV-seronegative subjects in any age group.

**Table 1:** Summarized γδ T cell profiles of study subjects

Age group	T cell subset	CMV-positive	CMV-negative	*P*-value (Mann–Whitney *U*-test)
21–40 years		(*n* = 39)	(*n* = 58)	
	Vδ2-negative	2·04% ± 0·3 (29·71 ± 5·75)	1·21% ± 0·08 (14·58 ± 1·5)	0·036 (0·009)
	Vδ2-positive	2·62% ± 0·37 (35·5 ± 6·4)	3·37% ± 0·38 (39·5 ± 4·7)	0·134 (0·385)
41–60 years		(*n* = 43)	(*n* = 40)	
	Vδ2-negative	2·44% ± 0·46 (40·14 ± 9·87)	0·85% ± 0·1 (11·42 ± 1·32)	< 0·0001 (0·0003)
	Vδ2-positive	2·17% ± 0·44 (29·62 ± 5·9)	2·44% ± 0·32 (34·8 ± 5·1)	0·085 (0·09)
61^+^ years		(*n* = 43)	(*n* = 32)	
	Vδ2-negative	3·67% ± 1·03 (58·16 ± 25·66)	0·7% ± 0·09 (7·01 ± 1·09)	0·0004 (< 0·0001)
	Vδ2-positive	2·06% ± 0·5 (44·1 ± 13·8)	3·07% ± 0·64 (43·7 ± 8·9)	0·09 (0·472)

Values in the CMV-positive and CMV-negative columns denote means and standard error for each subset as a percentage of total T cells and, in brackets, absolute numbers per μl of blood. CMV = cytomegalovirus.

### Identification of naive and memory γδ T cell subsets

**Fig. 2:**
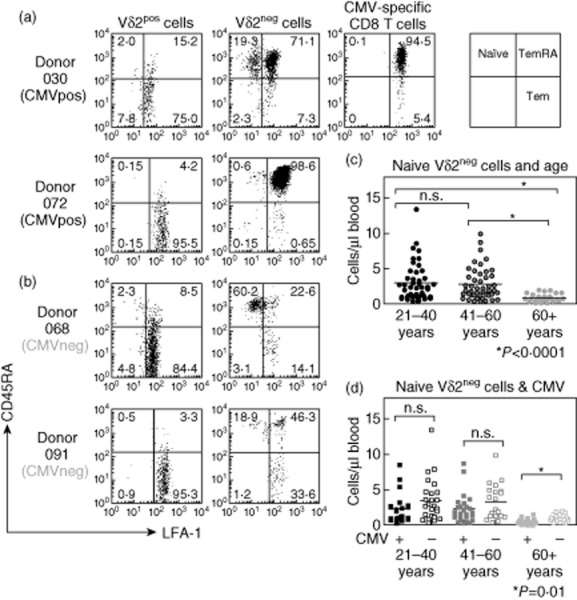
Effect of cytomegalovirus (CMV) carriage on naive and memory cell composition of γδ T cell subsets. Peripheral blood mononuclear cells (PBMC) were stained with T cell receptor (TCR)-γδ, Vδ2, lymphocyte function-associated antigen 1 (LFA-1) and CD45RA monoclonal antibody (mAb). Flow cytometry plots show LFA-1 (*x*-axes) *versus* CD45RA (*y*-axes) staining of Vδ2^pos^ and Vδ2^neg^ γδ T cell subsets in two CMV-seropositive donors (a) and two CMV-seronegative donors (b). Staining is shown on a logarithmic scale from 10^0^ to 10^4^ arbitrary units. LFA-1 *versus* CD45RA staining is also shown for CMV epitope-specific CD8^+^ T cells for one of the two CMV-seropositive donors by gating on human leucocyte antigen (HLA)-A1 (VTE) tetramer binding CD8^+^ T cells. Values indicate percentage of gated cells in each quadrant. Absolute numbers of naive Vδ2^neg^ γδ T cells were also compared between age groups (c) and between CMV-seropositive and CMV-seronegative (marked as ^+^ and – on *x*-axis, respectively) donors (d).

Total γδ T cells contain both naive (LFA-1^low^ CD45RA^high^) and memory cells (LFA-1^high^ CD45RA^high/low^) [19]. We thus determined whether naive and memory γδ T cell subsets were affected by CMV carriage in different age groups. Figure 2a,b shows representative examples of Vδ2^pos^ and Vδ2^neg^ γδ T cells in different donors. While Vδ2^pos^ cells were overwhelmingly CD45RA^low^ memory cells in both CMV-seropositive and seronegative donors, Vδ2^neg^ cells showed a distinct naive/memory profile which appeared to be linked to CMV status. In CMV-seropositive donors the Vδ2^neg^ γδ subset was skewed towards CD45RA^high^ revertant memory cells (denoted T_em_RA), very much like that observed for CMV-specific CD8 T cells. Overall, there was an increase in memory Vδ2^neg^ cells with age in CMV carriers, but this did not reach statistical significance (Supporting information, Fig. S2a). However, memory Vδ2^neg^ cells were reduced significantly in numbers as CMV-seronegative subjects became older (Supporting information, Fig. S2b).

**Fig. 3:**
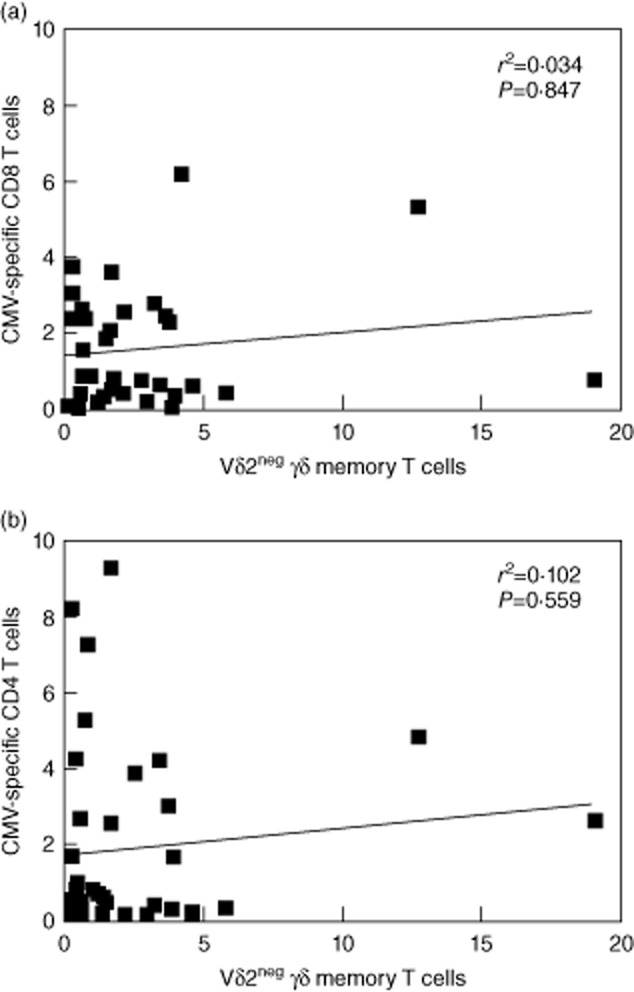
Comparison between Vδ2^neg^ γδ T-cell and cytomegalovirus (CMV)-specific αβ T cell frequencies in healthy donors. Charts show the correlation between Vδ2^neg^ γδ T cells and CMV-specific CD8^+^ T cells (a) and CMV-specific CD4^+^ T cells (b) in CMV-seropositive donors. Values on each axis denote the percentage of each cell type as a percentage of the total T cell repertoire. Significance (*P*) values at 95% confidence intervals are shown for each set of data. Correlations were determined using the non-parametric Spearman's rank test.

Further analysis showed that, independent of CMV status, there was a significant decrease in absolute numbers of naive cells in elderly donors (Fig. 2c) when compared with middle-aged and young donors (both *P* < 0·0001). CMV carriage associated with reduced naive Vδ2^neg^ cells in each group (Fig. 3d), but this only reached statistical significance in elderly donors (*P* = 0·01).

### Comparative analysis of Vδ2^neg^ γδ T cells with virus-specific CD4^+^ and CD8^+^ T cells

Although Vδ2^neg^ γδ T cells were higher in older population groups, there was considerable interindividual variation within all age groups. We questioned whether this variation was due to differences in frequencies of CMV-specific CD4^+^ and CMV-specific CD8^+^ T cells, both parameters also varying considerably between individuals in each group. CD4^+^ T cell frequency was based on IFN-γ responses against CMV lysate and CD8^+^ T cell responses were based on responses against a peptide cocktail representing six immunodominant antigens (IE-1, IE-2, pp65, pp50, gB, pp150), which would cover 90% of responders. This does not represent the complete CMV-specific T cell response, which could involve over 100 viral antigens [13]; however, this would be impractical to measure in a large cohort study such as ours. The results (Fig. 3) showed that frequencies of Vδ2^neg^ γδ T cells did not correlate with the CD8^+^ T cell response (*r*^2^ = 0·034; *P* = 0·847) or CD4^+^ T cell response (*r*^2^ = 0·102; *P* = 0·559). Some individuals had large Vδ2^neg^ γδ T cell expansions and weak CMV-specific CD8^+^/CD4^+^ T cell responses, some had strong CMV-specific CD8^+^/CD4^+^ T cell responses and low frequency of Vδ2^neg^ cells, and some had high levels of all subsets.

### Stability of γδ T cell subsets over time

**Fig. 4:**
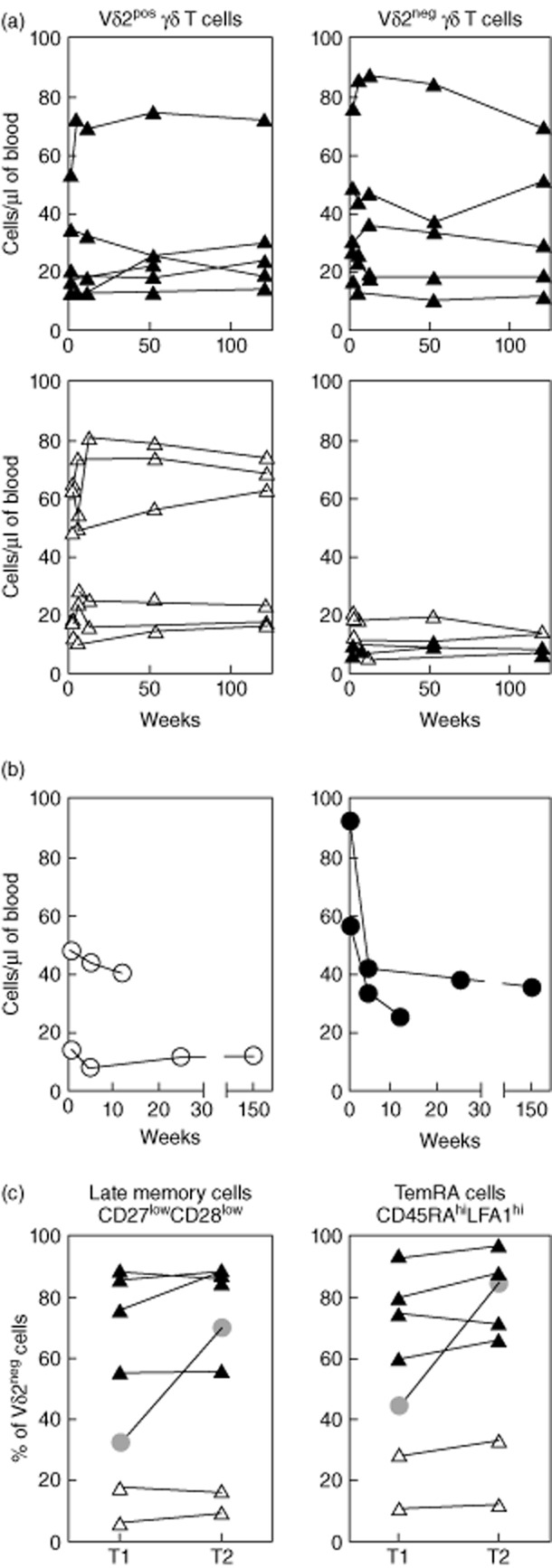
Dynamics of Vδ2^neg^ γδ T cells in healthy donors over time. Vδ2^pos^ and Vδ2^neg^ γδ T cell numbers were measured in a longitudinal analysis of six cytomegalovirus (CMV)-seropositive (top charts) and six CMV-seronegative healthy donors (bottom charts) (a; both groups composed of three young and three middle-aged subjects), and also in two immunocompetent subjects diagnosed with primary CMV infection (b; aged 38 and 43 years). Phenotypic changes in late memory and T-effector memory CD45RA-positive (T_em_RA) cells were also determined after a 12-month interval in six healthy donors (CMV-pos; black triangles, CMV-neg; white triangles) and one of the primary CMV patients (grey circle) (c). T1 represents 1 week post-diagnosis and T2 denotes 2 years post-diagnosis for the primary CMV samples. Values shown indicate the percentage of Vδ2^neg^ γδ T-cells expressing the given phenotype.

Herpesvirus-specific T cells are reported to fluctuate over time [29], so we were interested to learn if Vδ2^neg^ γδ T cells displayed this behaviour by performing longitudinal analysis on a random selection of six CMV-seropositive and six CMV-seronegative donors and two cases of primary CMV-infectious mononucleosis (IM) infection. Vδ2^neg^ cell numbers varied modestly in healthy donors over time (see Fig. 4a), mean 29·2% variation in CMV-seropositive and mean 35·7% in CMV-seronegative donors, while CMV-IM patients showed a more dramatic reduction in absolute number of Vδ2^neg^ γδ T cells in samples collected between 1 week post-diagnosis and subsequent time-points (Fig. 4b). All healthy donors showed very stable phenotypes at each time-point, but in CMV-IM Vδ2^neg^ γδ T cells were composed initially of both T_em_ and T_em_RA cells, and both CD27^high^CD28^low^ and CD27^low^CD28^low^ cells during the early phase of infection. However, after 2 years Vδ2^neg^ γδ T cells had shifted almost exclusively to the T_em_RA phenotype, with a concomitant shift to highly differentiated CD27^low^CD28^low^ cells (Fig. 4c). This change was more dramatic than those observed in healthy donors. In contrast, Vδ2^pos^ γδ T cells were composed mainly of less differentiated CD27^high^CD28^high^ CD45RA^neg^ central memory T cells (T_cm_), both during acute infection and 3 years later (data not shown).

### Effector memory phenotype and function of Vδ2^neg^ γδ T cells

**Fig. 5:**
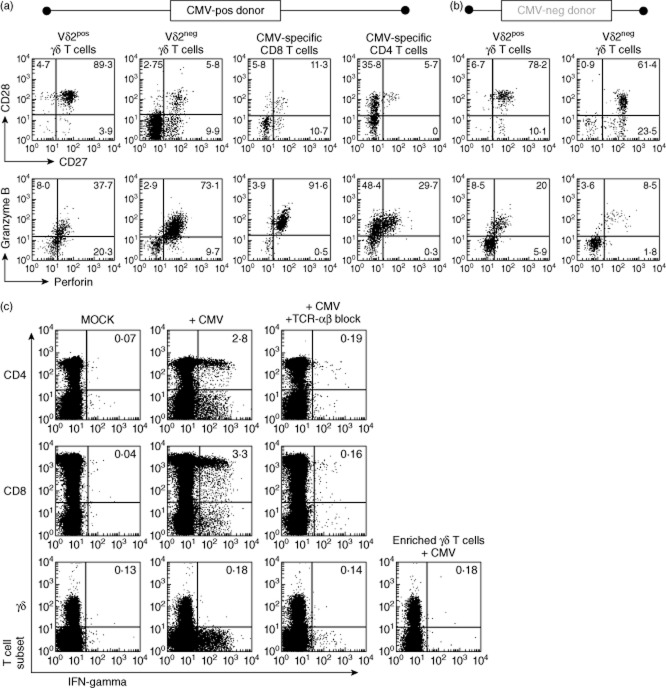
Vδ2^neg^ γδ T-cells share effector memory phenotype, but not *ex-vivo* effector function, with cytomegalovirus (CMV)-specific αβ T cells. Flow cytometry plots of CD27 *versus* CD28 and perforin *versus* granzyme B staining of gated cells from peripheral blood mononuclear cells (PBMC) of one CMV-seropositive (a) and one CMV-seronegative donor (b). The events shown are gated on T cell subsets indicated above each column of plots. For CMV-specific αβ T cells, events are gated on CMV tetramer (A1-VTE) binding CD8^+^ T cells and *ex-vivo* interferon (IFN)-γ-producing CD4^+^ T cells after 6 h stimulation with CMV lysate at 37°C. Effector function was tested by measuring cytokine production by different T cell subsets, including enriched γδ T cells (after depletion of αβ T cells), after co-incubation with partially human leucocyte antigen (HLA)-matched CMV-infected fibroblasts (c). Assays were carried out in the presence or absence of anti-T cell receptor (TCR)-αβ blocking antibodies. Plots are representative of four independent experiments. Stimulations with phorbol myristate acetate (PMA)/ionomycin were performed (not shown) to verify functional integrity of the different cell types in each experiment.

The above-described similarity in phenotype between Vδ2^neg^ γδ T cells and CMV-specific CD8 T cells prompted further comparative studies. Work by ourselves and others [5,6,30] has shown that CMV drives CD4^+^ and CD8^+^ T cells towards a highly differentiated T_em_ and T_em_RA phenotype. Because Vδ2^neg^ γδ T cells were increased in CMV carriers and, in common with CMV-specific CD4^+^ and CD8^+^ T cells, also appeared to increase with age, we hypothesized that these γδ T cells would also be driven towards a highly differentiated T_em_/T_em_RA phenotype. Figure 5 shows representative phenotypes of different T cell subsets in representative CMV-seropositive and CMV-seronegative donors. Vδ2^neg^ γδ T cells display remarkable similarity to CMV-specific CD8^+^ T cells in particular and CMV-specific CD4^+^ T cells to a lesser degree, but not to Vδ2^pos^ γδ T cells. Vδ2^neg^ γδ T cells in CMV-seropositive donors were composed of greater numbers of late memory CD27^low^CD28^low^ cells and contained higher levels of intracellular perforin and granzyme B than in CMV-seronegative donors, indicating greater differentiation and functionality of Vδ2^neg^ cells in terms of cytotoxicity. In contrast, Vδ2^neg^ γδ T cells in CMV-seronegative donors and Vδ2^pos^ γδ T cells in both sets of donors were composed mainly of less differentiated T_cm_ and T_em_ cells (Fig. 5a,b). This indicates that CMV carriage leads to a profound change in phenotype of Vδ2^neg^ γδ T cells, but not Vδ2^pos^ cells.

We tested freshly isolated Vδ2^neg^ γδ T cells, assumed to contain effector memory cells that were reactive against CMV-infected fibroblasts, for their ability to function in *ex vivo*. Figure 5c shows that fresh Vδ2^neg^ γδ T cells do not produce IFN-γ after co-incubation with CMV-infected targets. This was also the case when TNF-α production and CD107 degranulation was measured (data not shown), suggesting that Vδ2^neg^ γδ T cells do not possess immediate effector function to the same degree as CMV-specific αβ T cells in our assay system. It was possible that the extremely efficient recognition of infected targets by virus-specific CD4^+^ and CD8^+^ T cells masked the true potential of the γδ T cell response. However, this did not appear to be the case, as antibody blocking, or depletion, of CD4^+^ and CD8^+^ T cells had no enhancing effect on the γδ T cells in our *ex-vivo* assay.

**Fig. 6:**
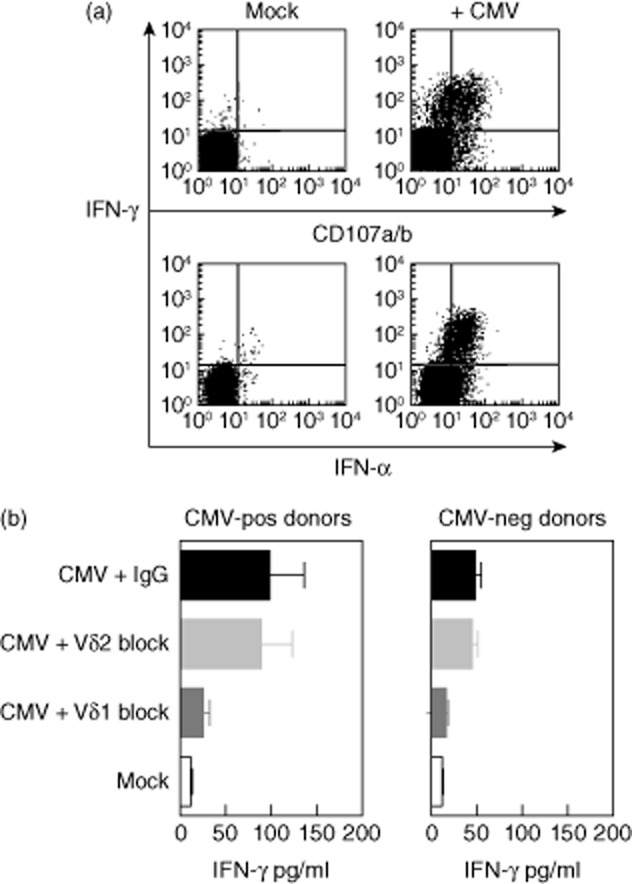
Recognition of virus-infected target cells by Vδ2^neg^ γδ T cells. *In-vitro* expanded γδ T cell lines tested for the ability to recognize cytomegalovirus (CMV)-infected (AD169 strain) human fibroblasts. Representative flow cytometry plots showing cytokine secretion and degranulation against CMV-infected targets from a CMV-seropositive donor (a). γδ T cell lines were also tested by interferon (IFN)-γ enzyme-linked immunosorbent assay (ELISA) after overnight incubation with CMV-infected stimulators (b). Data are pooled from independent experiments with T cell lines generated from three different CMV-seropositive and CMV-seronegative donors. Mock-infected targets were used as controls with anti-Vδ1, anti-Vδ2 and mouse immunoglobulin (Ig)G antibodies used to block recognition.

To confirm that Vδ2^neg^ γδ T cells had CMV-specific reactivity, we generated γδ T cell lines *in vitro* from CMV-seropositive and CMV-seronegative donors. Results show that T cell lines from both sets of donors, although at higher levels in CMV-seropositive cases, could produce cytokines (IFN-γ and TNF-α) and degranulate after co-incubation with CMV-infected fibroblasts, but not against mock-infected fibroblasts (Fig. 6a). This recognition could be blocked, either partially or completely, using the anti-Vδ1 monoclonal antibody but not with the anti-Vδ2 monoclonal antibody (Fig. 6b). This confirmed that Vδ2^neg^ γδ T cells in our donors were indeed reactive against CMV, with Vδ1^pos^ γδ T cells being a major component of this recognition.

## Discussion

CMV carriage in healthy humans is generally viewed as clinically benign, but it is clear that this relationship involves major perturbations in lymphocyte subsets over time [2,31,32]. This study is a detailed account of how γδ T cell subsets are skewed by the combined effects of CMV carriage and ageing in healthy individuals. In many older individuals we observed increased frequencies of Vδ2^neg^ γδ T cells, which were overwhelmingly of effector memory phenotype, a finding that mirrors the inflation of CMV-specific CD8^+^ effector T cells in elderly CMV carriers. The clinical relevance of this broad immune modulation by CMV is unclear, but is the subject of intense investigation.

While the increase in Vδ2^neg^ cells with ageing in CMV-seropositive donors was not statistically significant there was a significant decline in the Vδ2^neg^ cell frequency in CMV-seronegative donors, suggesting an intimate relationship between CMV carriage and the expansion and long-term maintenance of this presumed non-adaptive T cell subpopulation, as also shown by others while this paper was being prepared [33,34].

Vδ2^neg^ γδ T cell expansions, which were overwhelmingly Vδ1^pos^, exceeded 10% of total T cells in several middle-aged and elderly CMV-seropositive donors. As Vδ2^neg^ γδ T cells also display reactivity for tumour cells [25], immune responses against malignant cells *in vivo* may contribute towards these T cell expansions. However, the absence of such expansions in CMV-seronegative donors suggests that anti-tumour activity has a limited role.

CMV carriage was associated with reduced naive Vδ2^neg^ cell numbers in each age group, reaching significance in the elderly. However, naive Vδ2^neg^ γδ T cells were reduced more significantly in the elderly group as a whole, irrespective of CMV status. This finding may have also importance, as attrition in naive CD8^+^ T cells is linked with reduced immunity in old age [35].

While there was no pattern of correlation between frequencies of Vδ2^neg^ γδ T cells and virus-specific CD4^+^/CD8^+^ T cells, there was phenotypic similarity between these subsets, which are not shared by Vδ2^pos^ γδ T cells. In particular, Vδ2^neg^ γδ T cells were akin to CMV-specific CD8^+^ T cells; both are almost exclusively effector cells, including both T_em_ and T_em_RA cells, with a highly differentiated CD27^low^CD28^low^ phenotype. Vδ2^neg^ γδ T cells also expressed high levels of markers of cytotoxicity (perforin and granzyme B), similar to both CMV-specific CD8^+^ and CD4^+^ T cells. In contrast, Vδ2^pos^ γδ T cells were mainly CD45RA^low^ (CD45RO^high^), CD27^high^CD28^high^ and heterogeneous for cytotoxicity markers.

Highly differentiated Vδ2^neg^ γδ T cells in healthy people were very stable in number and phenotype over 3 years. However, the picture was more dynamic after primary infection. In the acute phase, the response was composed mainly of T_em_ (CD45RA^low^) and T_em_RA (CD45RA^high^) cells, but this response had rapidly contracted and shifted to an overwhelmingly T_em_RA phenotype with a concomitant shift towards end-stage highly differentiated cells. Conversely, no significant change in Vδ2^pos^ γδ T cell phenotype was observed. This analysis involved limited patient numbers, but the findings are consistent with those described in immunosuppressed CMV-infected transplant patients and CMV-infected newborns [23,24,26,33].

We confirmed Vδ2^neg^ γδ T cell reactivity against CMV-infected cells using *in-vitro*-expanded T cell lines. However, we could not demonstrate immediate effector activity using freshly isolated Vδ2^neg^ cells in *ex-vivo* assays, which was unexpected given the shared effector memory phenotype of Vδ2^neg^ γδ T cells and virus-specific αβ T cells. Being a distinct T cell lineage, γδ T cells may require an additional activation signal, but the observed result could also be a reflection of our experimental conditions; CMV-infected fibroblasts, while able to sensitize virus-specific CD4^+^ and CD8^+^ T cells, may not have expressed sufficient levels of the ligand(s) for optimal stimulation of freshly isolated Vδ2^neg^ γδ T cells. The use of non-autologous fibroblasts could also be problematic if stimulation occurs via an autologous non-MHC pathway. Another possibility is that Vδ2^neg^ γδ T cells are driven to exhaustion, as described for CMV-specific αβ T cells in elderly people [9,36,37] and CD8^+^ T cells with the CD28^low^/CD57^high^ phenotype [38,39]. Further work is necessary to test senescence in Vδ2^neg^ γδ T cells, although some caution is warranted when assessing function, as our experiments are based solely on circulating γδ T cells in the blood, and not from mucosal sites where Vδ2^neg^ γδ T cells are likely to be functionally more active. Of particular note, CMV-reactivity was also displayed using Vδ2^neg^ cell lines derived from CMV-seronegative donors. This state of readiness for expansion in CMV-seronegative donors provides support for these cells having a central role in the immune response after primary infection, and also highlights their potential in cellular immunotherapy of viral disease in CMV-seronegative patients.

Unlike for other T cell subsets, data on ligands for Vδ2^neg^ γδ T cells have been lacking, but this field is rapidly evolving. Willcox and colleagues have recently identified a stress ligand called endothelial protein C receptor, which is recognized by γδ TCR belonging to a Vδ5 T cell clone with dual CMV-specific and epithelial tumour-specific reactivity [40]. Furthermore, it has been shown that Vδ2^neg^ γδ T cells can be activated via CD16 engagement in a TCR-independent manner [41]. This may involve ligation via CMV-specific antibodies, as a positive correlation between anti-CMV IgG titres and T_em_ Vδ2^neg^ γδ T cells has been reported [34]. The discovery of additional ligands and activation pathways, especially for other more numerically dominant Vδ2^neg^ cells (namely Vδ1^pos^), is keenly awaited. It will be of interest to learn whether these ligands play a role in direct recognition of CMV-infected cells, indirect recognition of antigens cross-presented by professional antigen-presenting cells [42], recognition during latency [43] and after reactivation from latency. Ligand information will also be of immense value for CMV vaccine research [44], where the aim is to elicit broad multi-specific immunity [45]. Furthermore, it will be crucial to learn if Vδ2^neg^ γδ T cells are subject to immune-evasion by CMV, which is the case for other T cells and for natural killer (NK) cells [46].

To summarize, Vδ2^neg^ γδ T cells are increased in CMV carriers and particularly in more aged subjects. Given the reported shared reactivity of Vδ2^neg^ γδ T cells for CMV and epithelial tumour cells [25] and the greater incidence of cancer in the elderly, this finding may have great significance. Indeed, CMV seropositivity and increased numbers of Vδ2^neg^ γδ T cells coincide with lower risk of cancer in kidney transplant patients [47]. Further independent studies of CMV status and Vδ2^neg^ γδ T cells in cancer patients are thus a major priority for the future.

## Supporting Information

**Fig. S1.** Flow cytometry plots showing peripheral blood mononuclear cells (PBMC) stained with pan-T cell receptor (TCR)-γδ and anti-Vδ1 or anti-Vδ2 monoclonal antibodies from two cytomegalovirus (CMV)-seronegative (a) and two CMV-seropositive healthy donors (b). Values shown in the two upper quadrants of each plot indicate the percentage of lymphocytes staining for each γδ subset. **Fig. S2.** Analysis of memory Vδ2^neg^ γδ T-cells in cytomegalovirus (CMV)-seropositive (a) and CMV-seronegative donors (b) of different age groups. Charts show the absolute numbers of memory Vδ2^neg^ γδ T cells in subjects as determined by co-staining with CD45RA and lymphocyte function-associated antigen 1 (LFA-1). Memory cells comprised both T effector memory (T_em_) and T effector memory CD45RA-positive (T_em_RA) cells (see Fig. 2).
