# Supplementary material for: Cytomegalovirus drives Vδ2neg γδ T cell inflation in many healthy virus carriers with increasing age
Source: Clin Exp Immunol. 2014 Apr 24;176(3):418–28. doi: 10.1111/cei.12297 (PMC4008987; doi:10.1111/cei.12297)
Supplement: Supplementary file 1 [file cei0176-0418-sd1.ppt]

## Slide 1
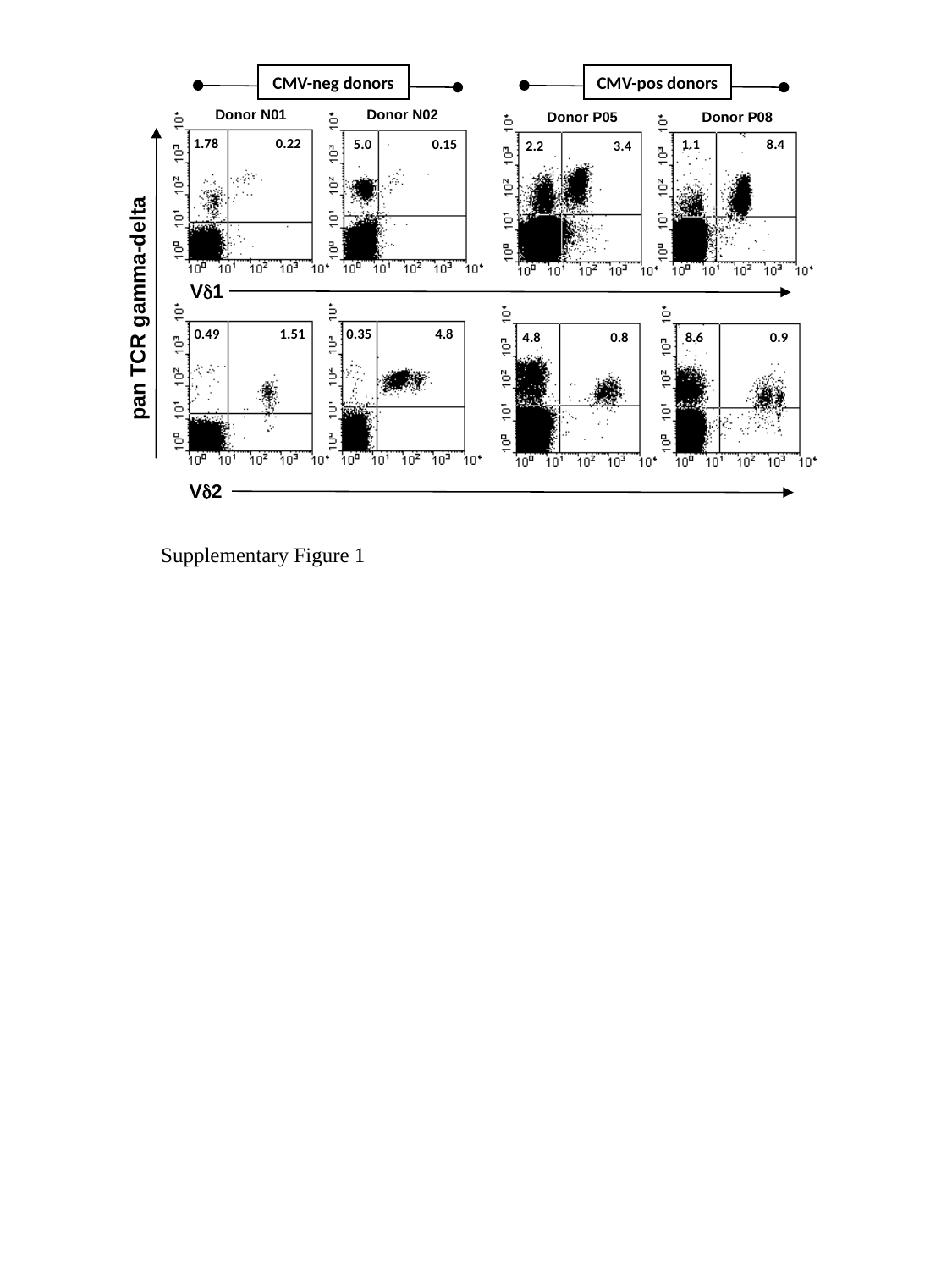

CMV-neg donors
CMV-pos donors
Donor N01 Donor N02
Donor P05 Donor P08
1.78 0.22
1.1 8.4
5.0 0.15
2.2 3.4
Vδ1
pan TCR gamma-delta
0.35 4.8
0.49 1.51
4.8 0.8
8.6 0.9
Vδ2
Supplementary Figure 1

## Slide 2
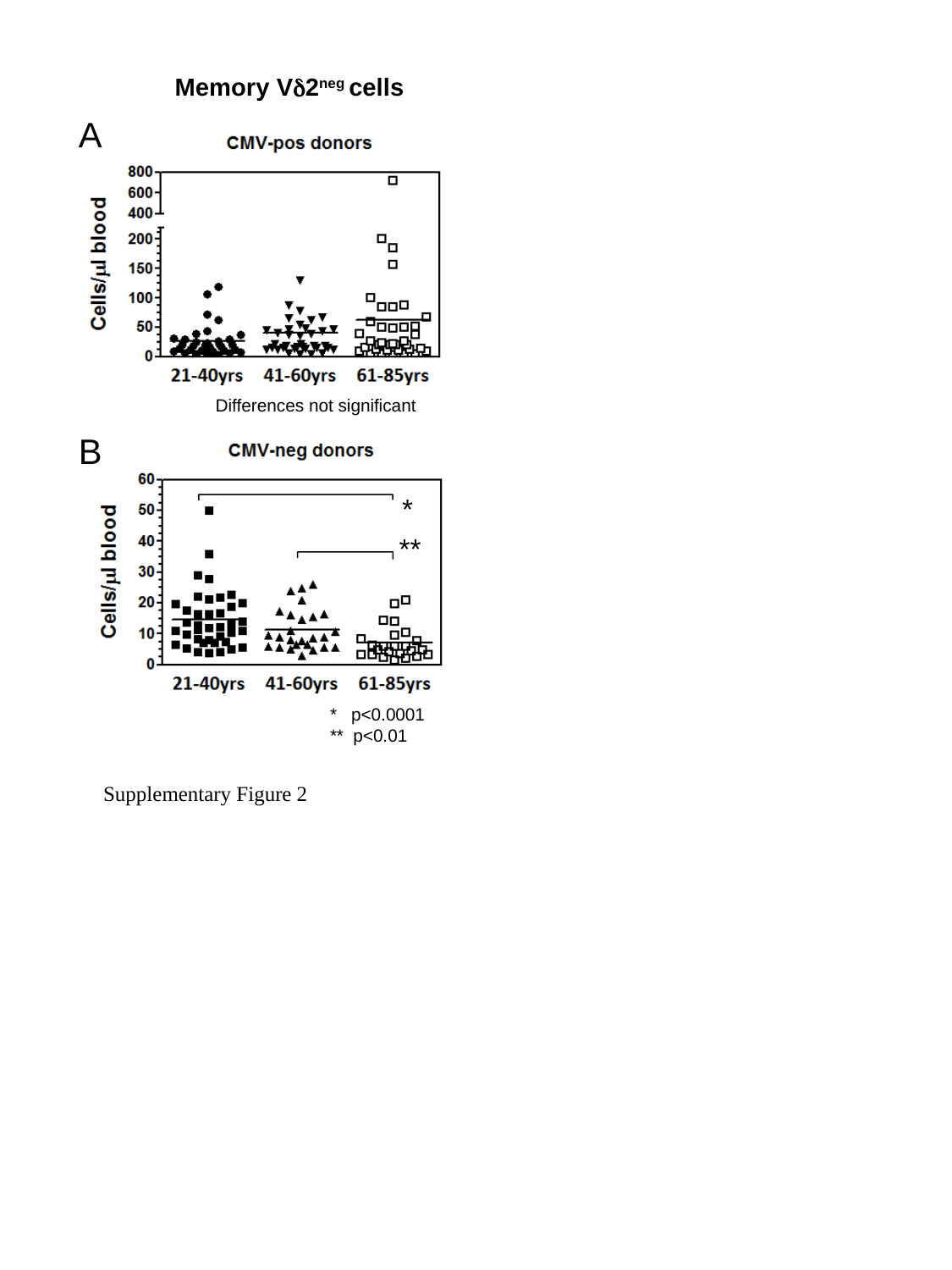

Memory Vδ2neg cells
A
Differences not significant
B
*
**
* p<0.0001
** p<0.01
Supplementary Figure 2
